# Dr. Adelbert John Douglass

**Published:** 1913-02

**Authors:** 


Dr. Adelbert John Douglass, L. I. C. Hospital, 1879, died at his home in Ileon, N. Y., in November, 1912.

